# *daf-41*/p23: A Small Protein Heating Up Lifespan Regulation

**DOI:** 10.1371/journal.pgen.1005188

**Published:** 2015-07-06

**Authors:** Klaus Richter

**Affiliations:** 1 Department Chemie, Technische Universität München, Garching, Germany; 2 Center for Integrated Protein Science Munich CIPS, Munich, Germany; Stanford University Medical Center, UNITED STATES

## *Dauer* and Life Span

The life span of non-renewing organisms is determined by the potential of their individual cells to maintain their functions while aging. Nematodes, like *Caenorhabditis elegans* with their 20 days of adult life, have proven to be excellent model systems to study organismal lifespan, its variability, and its regulation [1–3]. Early on, the life span could be linked to environmental conditions, like growth temperature and food intake [1,4]. In general, in these experiments, organisms develop and age slower and live longer at lower growth temperatures. This is evident from a clear relationship between temperature and life span ranging from 35 days to 9 days upon temperature changes from 10 to 25.5 degrees [1]. On the higher end of this temperature range, *C*. *elegans* can enter the *dauer* state, which is also found in response to starvation or the presence of *dauer* pheromone [5–7]. The formation of this stress-resistant state, which enables survival of the organism for longer than 3 months, requires morphological changes to the cuticule and inhibition of further development. Interestingly, it is entirely reversible without effects on the later adult life span [8]. This decision has been analyzed genetically in detail, identifying genes that promote *dauer* entry (DAF-c) and those that prevent *dauer* entry (DAF-d). These studies unravel the pathways, which cooperate in the decision whether to enter the path to the *dauer* state instead of normal development. The most prominent of those are the homologs of the insulin-like receptor DAF-2, the FOXO-transcription factor DAF-16, and the steroid hormone receptor DAF-12, amongst others [9–11]. The decision making requires the sensing of environmental factors and alteration of developmental programs in different tissues. Thus the number of genes influencing this decision is considerable.

Interestingly, several genes that control the entry into the stress- and starvation-resistant *dauer* state also exert control over the normal life span of the nematode [12,13]. Early aging markers include disorganization of muscular structure and reduction of pharyngeal activity and motility [14,15]. In this context, lower temperature, like some aging-related mutations, delays these early aging markers and likewise postpones later aging markers, like swallowing difficulties and general loss of motility. Despite knowing the individual function of many *dauer*-influencing genes, the reconstruction of regulated cellular pathways is complicated. This also originates from the fact that different cells are participating in the pathways as well as a contribution of humoral controls, implying that several individual cellular decisions culminate to regulate these pathways [16,17].

## *daf-41*/p23 Reprograms Temperature-Dependence in Aging

For many years it has been known that the cellular chaperone network is also contributing to these phenomena. In this context, the *daf-21* allele *p673*, representing a mutation in the heat shock protein 90, was known to cause constitutive dauer entry [18]. Moreover, the general regulator of the heat shock response HSF-1 was known to influence the life span in cooperation with other *dauer* genes and its depletion causes early onset of aging [19,20].

**Fig 1:**
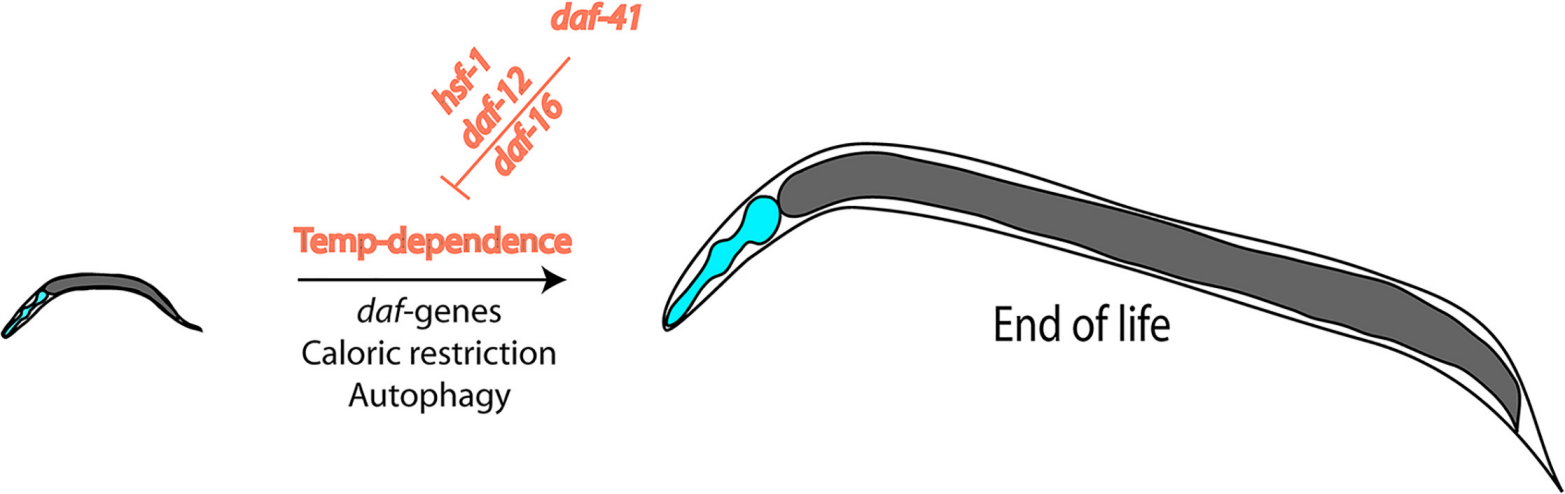
Nematode lifespan is regulated by temperature amongst other influences. This temperature dependence is the result of transcriptional networks and DAF-41/p23, which influences the transcriptional activities of HSF-1, DAF-12, and DAF-16.

In this issue of *PLOS Genetics*, Horikawa and coworkers address critical questions at the crossroads of stress-resistance, longevity, and chaperone involvement by investigating a deletion mutant of the cochaperone p23, an effector protein of Hsp90. They first determine that the deletion strain constitutively enters *dauer* and name the gene *daf-41*. They find that the usual temperature-dependence of the lifespan is altered in this strain with a much smaller temperature-influence than known for the wild-type background (Fig 1). This makes the deletion strain short lived at low temperatures and long lived at high temperatures. While it has been thought up until recently that the slow development and aging at low temperatures reflects the slower turnover of metabolites and the slower rate of all biochemical processes based on plain physical principles, being able to influence this effect by genetic means implies the existence of a biological control. Recent reports had suggested that such programs may exist [21]. Also, temperature-sensitive neurons had been reported to influence the aging process, similar to the findings reported here [22]. In general, these studies show that development does not necessarily has to be slow at low temperatures and fast at high temperatures, and, importantly, with *daf-41* a regulator is uncovered that influences this program.

Horikawa and coworkers also provide information on the mechanism—how such a response may be regulated by addressing the transcriptional networks influenced by the *daf-41* mutation. They show that the deletion of p23 influences several transcriptional outputs. At low temperatures, the influence on the steroid hormone receptor DAF-12 and on DAF-16 is especially relevant. At high temperatures, instead, the lifespan regulation originates from influences on the activity of the heat-shock factor HSF-1, making the stress response stronger in the absence of the Hsp90 cofactor. Thus, p23 appears to balance the transcriptional responses of these three transcription factors relevant for *dauer* formation, longevity, and aging, and in this way enables control over these processes at different temperatures.

## DAF-41/p23: How Can it Work?

Temperature-dependent growth is observed in all organisms, from bacteria to metazoa, and generally has been attributed to differences in metabolic rates. The recent observations now cast in doubt the general belief that plain physics controls the temperature-dependent effects on life span, as at least metazoa appear to have developed a program that controls these growth rates based on transcriptional networks. It is thus very exciting to see that another potentially deterministic program controls the progression of *C*. *elegans* through its larval stages to adulthood and during aging and adjusts the growth rate to the environmental conditions.

While the molecular details at the basis of this regulation are still elusive, it is worthwhile to look at the known biochemical functions of p23 and its interaction partner Hsp90. Hsp90 is known to regulate transcriptional outputs by influencing the activity of dozens of transcription factors via their cellular stability. p23, likewise, has been shown to influence the activity of transcription factors, including steroid hormone receptors and HSF-1 [23]. In several cases p23 and Hsp90 cooperate, but also individual activities have been observed for p23 [24]. The biochemical and structural aspects of their interaction are well studied, including by structural characterization of the Hsp90-p23 complex [25]. p23 binds to a conformational state of Hsp90 adopted during its ATPase cycle. Specifically, it recognizes an ATP-induced N-terminal dimerized conformation, which is populated just prior to ATP hydrolysis and remains bound to Hsp90 during the hydrolysis reaction. It controls the hydrolysis rate and enables stable complexes between Hsp90 and its protein clients. Processed client proteins are released after ATP hydrolysis. Essentially, this characteristic behavior contributed to the identification of the protein p23 more than 20 years ago, when it was uncovered as part of the protein assemblies involved in steroid hormone receptor maturation in mammals [26,27]. The influence of Hsp90 and p23 on their clients is not fully understood and rarely has a combinatorial effect on several clients being studied. However, tinkering with Hsp90 inhibition leads to many different phenotypic traits in flies [28], showing that in other model systems, several signaling networks are influenced simultaneously.

With the study of Horikawa and coworkers, this chaperone machinery now also moves into the center of life span regulation. Previously Hsp90, like p23, was found to regulate Hsf1 activity [29], and its depletion strongly induces the heat-shock response [30]. The chaperones’ involvement in the cellular folding process of transcription factors was, until now, seen as a contribution to the regulation of individual transcription factors. The new picture emerging is that by regulating simultaneously the activity of several transcriptional outputs, p23 enables complex developmental decision making. The present study thus integrates the cellular chaperone network surrounding the molecular chaperone Hsp90/DAF-21 into the complex decision making to enter the *dauer* state and to determine the organismal life span.
